# Effect of educational program based on theory of planned behavior on osteoporosis preventive behaviors: a randomized clinical trial

**DOI:** 10.1186/s12891-021-04861-x

**Published:** 2021-11-23

**Authors:** Nasim Pakyar, Sarieh Poortaghi, Shahzad Pashaeypoor, Farshad Sharifi

**Affiliations:** 1 Nursing and Midwifery Care Research Center, School of Nursing and Midwifery, Tehran University of Medical Sciences, Tehran, Iran; 2 Department of Community Health and Geriatric Nursing, School of Nursing and Midwifery, Tehran University of Medical Sciences, Tehran, Iran; 3 Elderly Health Research Center, Endocrinology and Metabolism Population Sciences Institute, Tehran University of Medical Sciences, Tehran, Iran

**Keywords:** Chronic disease, Theory of planned behavior, Health education, Osteoporosis

## Abstract

**Background:**

The prevalence of chronic diseases is increasing worldwide. Implementing educational programs is an important step in prevention of chronic diseases in the community setting. This study was conducted to assess the effect of educational program based on the Theory of Planned Behavior (TPB) on the osteoporosis preventive behaviors in middle-aged individuals.

**Methods:**

A randomized clinical trial was conducted on 64 middle-aged individuals presenting to primary care centers. A researcher-made questionnaire developed according to “a guide for compiling and analyzing the questionnaire based on TPB” was used for data collection. Random block sampling was applied to assign participants to control and intervention groups after ensuring the validity and reliability. An educational program on osteoporosis prevention was conducted in six educational sessions based on the TPB constructs for the intervention group in primary care centers. Control group received routine education about lifestyle changes including osteoporosis by primary care centers. Eight weeks after the intervention, the questionnaires were completed again and the data were analyzed using the SPSS V16 software.

**Results:**

Independent t-test found no significant difference in the mean score of knowledge, osteoporosis preventive behavior, attitude, subjective norms, perceived behavioral control and behavioral intention between intervention and control groups before intervention (*p* > 0.05). After the intervention, however, a significant difference was found in the mean score of knowledge, attitude, subjective norms, perceived behavioral control and behavioral intention between the cases in intervention and control groups (*P* < 0.05). In addition, based on repeated measurement ANOVA, the intervention had a significant effect on knowledge, preventive behaviors, attitude, subjective norms, perceived behavioral control, and behavioral intention (*P* < 0.05).

**Conclusions:**

The results of the present study showed that implementation of an educational intervention based on the Theory of Planned Behavior significantly increased the knowledge along with all constructs of TPB in osteoporosis preventive behaviors.

**Trial registration:**

This study was registered in the Iranian Registry of Clinical Trials IRCT2017081735647N2 (11/10/2017).

## Background

We are facing a marked increase in chronic diseases in all countries, especially developing countries, as a result of increased life expectancy, obesity, unhealthy nutrition, and low physical activity. One of these highly prevalent chronic diseases is osteoporosis [1, 2]. The World Health Organization (WHO) selected the theme “love your bones and protect your future” for the international day of osteoporosis in 2017, which emphasizes the importance of familiarity with osteoporosis and its preventive strategies [3].

According to the studies investigating osteoporosis in different parts of the world, about 75 million people suffer from this disease in Europe, Japan, and the USA [4]. This statistic is very similar to that of heart disease [5, 6], indicated by the fact that in 2014 more than 10 million American individuals were osteoporotic and 34 million people suffered from bone loss [7]. According to a report from the Rheumatology Research Center of Tehran University of Medical Sciences, six million individuals and two million postmenopausal women had osteoporosis in Iran in 2010. In addition, 50% of men and 70% of women above 50 years of age experience osteoporosis or bone loss [8]. If effective preventive measures are not implemented, it is predicted that the global cost of osteoporosis may reach 200 billion dollars by 2040. Despite the increasing prevalence of osteoporosis in Iran, there is insufficient information about its true prevalence and the associated burden.

According to previous studies, about 80 factors have been identified to cause osteoporosis of which 15% have a larger impact on this disease [9]. Sex, gender, skeleton size, alcohol, caffeine and tobacco consumption, reduced estrogen, premature menopause before the age of 45, reduced calcium intake, and physical immobility are the main causes of this disease. Furthermore, family history of fractures, history of using glucocorticoids for more than six months, hereditary diseases, Cushing’s syndrome, hyperthyroidism, and mal-absorption syndrome are secondary causes of osteoporosis [10–12]. Furthermore, reduced intake of milk and dairy products as well as low physical activity affect bone density [13, 14]. Given the fact that the prevention is prioritized over treatment, the need for osteoporosis prevention is a necessity. Osteoporosis prevention strategies include maximizing bone mass and minimizing the process of reducing bone density through health education and health promotion programs [13, 15]. It has been estimated that 20–50% of the bone density changes are modifiable because they are related to lifestyle [15–18]. Studies have shown that physical activity and adequate calcium consumption are very crucial in preventing osteoporosis [19]. In educational interventions, an important step is to select a model or theory based on the nature of the problem; moreover, the effectiveness and objectives of the model or theory should be consistent with the goal of the intervention program. Health education without a plan is a failed or ineffective attempt [20]. The impact of interventions on health outcomes is highly related to initiate and maintain health behavior change [21]. Hence choosing an appropriate educational model helps to start the program and keep it in the right path. The more suitable the theoretic support for the health needs, the more effective the health educational programs will be [22]. Applying theories for osteoporosis prevention education can help identify areas that need additional emphasis within programs [23].

TPB provides a useful framework to explain and predict health behaviors. This theory was introduced in 1885 and developed in 1980 by Fishbein & Ajzen [24]. The backbone of this theory is that the intention of the individuals to undertake a certain behavior is a predictive factor for that behavior. The intention itself has been realized under the effect of attitude towards the behavior plus subjective norms and behavioral control [25].

Attitude reflects the positive and negative evaluation of a behavior and consists of two subsections, including behavioral beliefs and evaluating the results of the behavior. Subjective norms is another construct of this theory, which refers to social pressure known by the individual to perform or ignore the intended behavior and is determined by ordering beliefs (by the society). The third section is perceived behavioral control, which refers to the degree of an individual’s feeling about the extent of doing or not doing a behavior under their control. Control factors consist of internal and external elements. Internal factors are related to the individual and include the skills, abilities, information, and feelings, while external factors refer to factors such as environmental and occupational factors. Behavioral intention expresses the intensity of an individual’s intent to undertake the intended behavior. Behavioral intention always precedes and is related to behavior [26, 27]. According to Ajzen et al., one of the most important determining factors is the individuals’ behavioral intention, which results in doing a healthy behavior. The effect of attitude and others’ expectations such as family, friends, etc. on adopting a health behavior can be seen in the subjective norms section [28–30]. Therefore, TPB was used in this study due to the importance of preventive behaviors in osteoporosis prevention.

Community health nurses, as a key members in health education, can streamline informed decision-making by educating the middle-aged population about osteoporosis prevention. They can play an important role in promoting health behaviors aiming at disease prevention and reduction of treatment costs imposed on patients and health systems. Health programs aiming at improving the health of the middle-aged population including periodic evaluation of their health (health services offered to Iranian women and men) are currently implemented with the aim of health improvement, disease prevention, and lifestyle modification to smoothen the transition to middle age. Given the increasing prevalence of osteoporosis in Iran and the effective role of preventive measures, it is necessary to prevent this disease by educational interventions based on suitable models. Considering that osteoporosis has been identified as an important priority in Iran and the world, and since the populations covered by primary care centers only receive routine education about lifestyle change including osteoporosis, implementing a well-designed and comprehensive educational program is an important step in planning educational interventions and improving preventive behaviors.

### Purpose

To assess the effect of an educational program based on the Theory of Planned Behavior on the osteoporosis preventive behaviors in middle-aged individuals.

## Method

This randomized clinical trial study was conducted to 64 middle-aged individuals (30–59 years old) presenting to primary care centers affiliated to Kermanshah University of Medical Sciences, Iran. According to previous studies, assuming an error level of 0.05, test power of 80%, and loss to follow-up of 20% in each group, it was calculated that 34 subjects were required in each groups, and a total of 68 subjects were recruited. Preventive behaviour proxied by 95% confidence and 80% power, with expected mean difference between groups 4 points was considered as the main outcome for calculating sample size. After obtaining clearance from the Research Ethics Committee, one primary care center center (among 25 centers) was selected randomly and all the participants were selected from this center. This center provided health services to 4515 middle-aged women from whom the participants were selected. The inclusion criteria were willingness to participate in the study, age 30–59 years, ability to speak, perceive, and learn, negative history of osteoporosis, negative history of attending similar educational sessions. After this phase, informed consent was obtained from the participants. The exclusion criteria were reluctance to continue the study and missing two educational sessions in a row. Random block sampling (including 17 blocks of four sequences (was used to allocate the participants to intervention and control groups. Randomization sequence maintained by the statistician who was not involved in conducting the intervention in the field before the pre-test. Sealed dark envelopes were used for concealment. Pre-test was conducted for both groups using a researcher-made questionnaire that were completed independently by the participants. This questionnaire was developed according to recommendations for “Constructing a Theory of Planned Behavior Questionnaire” [31] using valid and reliable literature. The first section consisted of 18 questions including demographic questions (age, sex, employment, marital status, education level, income, insurance, number of deliveries and family members, BMI and past medical and drug history). The second section included 14 questions related to the level of knowledge about osteoporosis (signs and symptoms, risk factors, prompt diagnosis methods of osteoporosis, calcium intake through diet, common complications). In this section, a correct answer scored two points, “I do not know” scored one point, and an incorrect answer scored zero points. The total score of this section ranged between 0 and 28 with a higher score indicating more knowledge. The third part consisted of 12 questions addressing preventive behaviors of osteoporosis (physical activity, consumption of calcium-rich foods, and avoiding smoking and caffeinated drinks); the total score of this section ranged between 1 and 60 with a higher score indicating better preventive behaviors. The fourth part of the questionnaire was related to the TPB constructs including questions about attitude toward osteoporosis prevention behaviors (9 questions, score range: 1 to 45), perceived behavioral control (8 questions, score range: 1 to 40), subjective norms (4 questions, score range: 1 to 20), and intention to carry out osteoporosis prevention (7 questions, score range: 1 to 35). These questions were extracted by reviewing the relevant literature and categorized according to a five-point Likert scale (Strongly disagree = 1, Disagree = 2, Neither agree nor disagree = 3 Agree = 4, Strongly agree = 5) with a higher score representing proper osteoporosis preventive behavior.

The content and face validity of the questionnaire was approved by 10 experts in this field (See Related File: Questionnaire Psychometric data). The reliability of the questionnaire was checked by internal consistency method and the results showed a Cronbach’s alpha of 0.87, which was acceptable.

Control group received routine education about lifestyle changes including osteoporosis by primary care centers. For intervention group, an educational program designed based on TPB constructs was presented in six one-hour sessions, two sessions per week (overall 3 weeks) using different interactive methods like group discussion, and question and answer. The educational topics included comprehensive information about osteoporosis, its side effects, prevalence, determinants, and preventive behaviors with emphasis on lifestyle. In addition, TPB constructs were discussed in the educational sessions in an interactive manner. The contents of the educational sessions were collected using creditable literature and was compatible with culture, regional habits and context. This means that in compiling this content, the living conditions of the participants and their preferences were taken into account. Validity of educational content approved by five experts in this field before conducting the intervention (See Related File: Questionnaire Psychometric data).

**Fig. 1:**
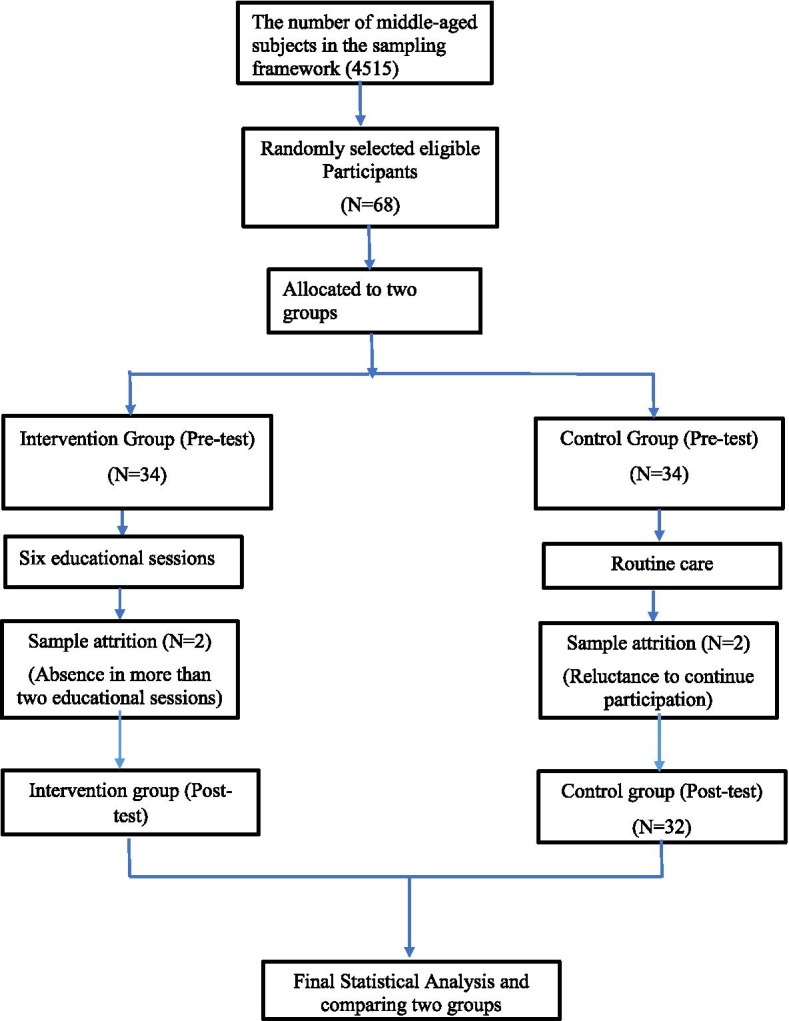
Consort diagram of study

At the end of each session, the presented material was given to the participants and their families in a written format. Eight weeks after the intervention, a posttest was administrated to both groups and the results were compared considering the purpose of the study. For ethical reasons, a handbook containing the educational material was given to the participants in the control group after the post-test  (Fig. 1).

The SPSS software version 16 was used for data analysis. The main analytical method for this study was the intention-to-treat analysis including all randomized participants regardless of loss to follow-up. Therefore, there were missing values in the control and intervention groups in analyses. Descriptive statistics are presented as frequency, central indices, and standard deviation in the form of tables. Chi -square test, Fisher’s exact test, and independent samples t-test were used for data analysis. The significance level was set at 0.05 for all tests. The Kolmogorov-Smirnov test was applied to assess data normality. Homogeneity of the research participants in intervention and control groups was evaluated before the study. Chi-square was used for qualitative variables and t-test was used for quantitative normal variables to investigate homogeneity and test the research hypotheses. Independent t-test was used to evaluate the difference in the mean scores of the questionnaires between the two groups before and after the intervention. Repeated measures analysis of variance was used to determine the effect of intervention on the mean score of knowledge, prevention behaviors, attitude, subjective norms, perceived behavioral control and behavioral intention. This study was conducted based on the following hypotheses “Educational program based on theory of planned behavior is effective on osteoporosis prevention behaviors in middle-aged people. It should be noted that this study was the results of an MSc thesis approved by the Ethics Committee of Tehran University of Medical Sciences (IR.TUMS.FNM.REC.1396.2693). The study was registered in the Iranian Registry of Clinical Trials (IRCT2017081735647N2) (11.10.2017).

## Results

In this study, 64 middle-aged participants aged 30–59 years old were assigned to intervention (n = 32) and control (n = 32) groups. Table 1 shows the relative and absolute frequency of participants in the study in terms of demographic variables. According to the demographic data, 57.4% of the participants were women, 45.6% were housewives, 82.4% were married, and 30.9% had a university education. Only 26.5% of the respondents considered their income as sufficient. Moreover, 26.5% had a medical history and 75% had health insurance. According to chi-square and Fisher’s exact tests, there was no significant difference in the variables between two groups; in other words, the groups were similar in terms of the above variables (*P* > 0.05).

**Table 1:** Distribution of relative and absolute frequency of demographic variables in the Intervention and control groups

Variable	Intervention	control	Total	Test statistic	*P*-value
	N (%)	N (%)	N (%)		
Sex					
Male	14(48.3)	15(51.7)	29(42.6)	0.06	0.806
Female	20(51.3)	19(48.7)	39(57.4)		
Employment					
Housewife	16(51.6)	15(48.4)	31(45.6)	7.06	0.216
Employee	4(28.6)	10(71.4)	14(20.6)		
Free	9(69.2)	4(30.8)	12(19.1)		
Retired	2(66.7)	1(33.3)	2(4.4)		
Unemployed	0(0)	2(100)	7(2.9)		
Others	3(60)	1(33.3)	5(7.4)		
Educational level					
Under the diploma	9(52.9)	8(47.10	17(25)	0.25	0.969
Diploma	11(52.4)	10(47.60	21(30.9)		
Associate Degree	9(47.4)	10(52.6)	19(27.9)		
Masters	5(45.5)	6(54.5)	11(16.2)		
Marital status					
Single	4(50)	4(50)	4(50)	1.405	0.704
Married	27(48.2)	29(51.8)	56(82.4)		
Divorced	1(100)	0(0)	1(1.5)		
Widow	2(66.7)	1(33.3)	3(4.4)		
Sufficient income					
Yes	10(55.6)	8(44.4)	18(26.5)	0.339	0.844
No	5(54.5)	6(54.5)	11(16.2)		
Somewhat	19(48.7)	20(51.3)	39(57.4)		
Physical illness					
Yes	11(61.1)	7(38.9)	18(26.5)	1.209	0.272
No	23(46)	27(54)	50(73.5)		
Insurance					
Has it	23(45.1)	28(54.9)	51(75)	1.961	0.161
Does not have	11(64.7)	6(35.3)	17(25)		
Number of Deliveries					
≤2	14(48.3)	15(51.7)	29()	0.409	0.394
>2	6(60)	4(40)	10()		
Number of family members					
≤3	18(47.4)	20(52.6)	38(55.9)	0.239	0.625
>3	16(53.3)	14(46.7)	30(44.1)		
Family history of osteoporosis					
Yes	5(14.70)	5(14.70)	10(14.70)	2	0.491
No	19(55.84)	22(64.71)	41(60.30)		
Don’t Know	10(29.41)	7(20.58)	17(25)		
Sun exposure					
Under direct sunlight	14(41.17)	15(41.11)	29(42.65)	1	0.806
From behind the window	20(58.82)	19(55.88)	39(57.35)		
Excessive consumption of coffee and tea					
Yes	15(44.11)	18(52.94)	33(48.53)	1	0.467
No	19(55.88)	16(47.05)	35(51.47)		
Smoking					
Yes	5(14.70)	6(1.64)	11(16.17)	1	0.742
No	29(85.29)	28(82.35)	57(83.33)		
Age	41.1 ± 8.3	36.7 ± 7.1	38.9 ± 8.01	2.37	0.02
BMI	26.2 ± 2.7	26.6 ± 4.1	26.6 ± 3.5	0.029	0.977

The mean age of the subjects was 38.9 ± 8 years. There was a significant difference in age between the two groups (*P* = 0.02). Therefore, the effect of this variable was controlled as a confounder. The mean body mass index was 26.6 ± 3.5. There was no statistically significant difference in body mass index between the two groups (*P* > 0.05) (Table 1).

Based on the statistical analysis, there was a significant difference in the mean scores of knowledge, prevention behaviors, attitude, subjective norms, perceived behavioral control and behavioral intention before and after the intervention in the intervention group (*P* > 0.05). There was no significant difference between the mean of the mentioned variables in the control group before and after the intervention (*P* > 0.05) (Table 2).

**Table 2:** Mean and standard deviation of knowledge scores, prevention behaviors, attitude, mental norms, perceived behavioral control and behavioral intention in the intervention and control group before and after intervention

Variables	Group	The level	Mean ± Sd	t	*P*-Value^*^	Mean Diffrence	T	*P*-Value^**^
Knowledge	Intervention	PreTest	18.06 ± 4.54	−8.46	0.001	6.7 ± 4.48	7.93	0.001
		Post Test	24.7 ± 2.19					
	Control	PreTest	20.9 ± 3.7	0.524	0.604	−0.187 ± 2.02		
		Post Test	20.7 ± 3.4					
Prevention behaviors	Intervention	PreTest	37.9 ± 5.6	−5.8	0.001	5.6 ± 5.5	5.94	0.001
		Post Test	43.5 ± 4.5					
	Control	Pretest	41.9 ± 3.9	1.247	0.222	−0.281 ± 1.2		
		Post Test	41.6 ± 3.9					
Attitude	Intervention	Pretest	33.4 ± 3.9	−8.23	0.001	7.28 ± 5	8.09	0.001
		Post Test	40.7 ± 4.6					
	Control	Pretest	35.2 ± 3.7	0.432	0.669	−0.093 ± 1.2		
		Post Test	35.09 ± 3.7					
Subjective norms	Intervention	Pretest	13.3 ± 3.02	−7.6	0.001	4.2 ± 3.1	6.5	0.001
		Post Test	17.5 ± 2.3					
	Control	Pretest	12.5 ± 2.47	2.272	0.060	0.406 ± 1.01		
		Post Test	12.9 ± 2.2					
Perceived behavioral control	Intervention	Pretest	29.3 ± 2.45	−6.9	0.001	5.5 ± 4.5	6.8	0.001
		Post Test	34.8 ± 3.81					
	Control	Pretest	30.1 ± 2.8	0.533	0.598	−0.098 ± 0.99		
		Post Test	30.03 ± 2.7					
Behavioral intention	Intervention	Pretest	25.1 ± 3.3	−6.7	0.001	5.6 ± 4.7	33.9	0.001
		Post Test	30.8 ± 4.3					
	Control	Pretest	27.2 ± 3.7	−1.063	0.296	0.187 ± 0.99		
		Post Test	27.3 ± 3.3					

^*^ Paired T Test

^**^ Independent Samples Test

Repeated measures analysis of variance showed that the effect of intervention was significant. In addition, the interaction between the knowledge score, prevention behavior, attitude, subjective norms, perceived behavioral control and behavioral intention and group showed a significant difference in the process of change. Furthermore, there was a behavioral difference between the two groups during the study (*P* < 0.05).

As shown in Table 3, the probability of accepting the null hypothesis for comparison of posttest scores between intervention and control groups was less than 0.05 for the constructs of attitude, subjective norms and behavioral control. In other words, after adjusting the difference in the pretest scores of attitude, subjective norms, and behavioral control between case and control groups, there was still a significant difference in the posttest score between the two groups (*P* < 0.05). However, there was no significant difference in the knowledge scores, prevention behaviors and behavioral intention (*p* > 0.05) (Table 3).

**Table 3:** The results of variance analysis of repeated measures on knowledge scores, prevention behaviors, attitude, mental norms, perceived behavioral control and behavioral intention in the control and intervention groups

Variable	Source	Sum of Squares	df	Mean Square	F	*P*-Value
Knowledge	Knowledge	341.2	1	341.2	56.3	0.001
	Knowledge* group	381.5	1	381.5	56.3	0.001
	Group	11.8	1	11.8	0.606	0.439
	Error	1214.8	62	19.5		
Preventive behaviors	Preventive behaviors	231.1	1	231.1	28.9	0.001
	Preventive behaviors * group	282.03	1	282.03	35.3	0.001
	Group	34.03	1	34.03	1.01	0.318
	Error	2081.9	62	33.5		
Attitude	Attitude	413.2	1	413.2	62.2	0.001
	Attitude * group	435.1	1	413.2	65.5	0.001
	Group	116.2	1	116.2	4.5	0.037
	Error	1584.5	62	25.5		
Subjective norms	Subjective norms	171.1	1	171.1	42.7	0.001
	Subjective norms * group	116.2	1	116.2	42.7	0.001
	Group	236.5	1	236.5	23.3	0.001
	Error	628.9	62	10.1		
Perceived behavioral control	Perceived behavioral control	239.2	1	239.2	44.2	0.001
	Perceived behavioral control * group	255.9	1	255.9	47.3	0.001
	Group	126	1	126	9.94	0.002
	Error	785.8	62	5.4		
Behavioral intention	Behavioral intention	276.1	1	276.1	46.9	0.001
	Behavioral intention * group	242	1	242	41.1	0.001
	Group	15.12	1	15.1	0.702	0.405
	Error	1335.8	62	21.5		

* interaction between each variable with group (intervention or control)

In order to address the effect of four participants that were lost to follow-up on the main results of the study, sensitivity analysis was performed for each domain. According to the group each case was allocated to, the mean change of the scores was calculated and the analyses were performed again. The statistical significance did not change in any domain.

## Discussion

In this study, the effect of educational program based on the Theory of Planned Behavior on osteoporosis preventive behaviors of middle-aged people was assessed according to the research hypotheses and the following results were obtained. The results of the analysis of demographic variables and disease data showed no significant differences between the two groups before the intervention, except for age whose effect was controlled using ANCOVA. In other words, the two groups were homogeneous in terms of demographic variables.

Independent sample t-test showed no significant differences in osteoporosis preventive behaviors between the two groups before the intervention. After the intervention, however, a significant difference was found in osteoporosis preventive behaviors between the two groups (*p* = 0.001). This is consistent with the results of a study which used theories of Health Belief Model and Theory of Reasoned Action for osteoporosis prevention education. Results of this study showed statistically significant improvements for the Health Belief Model constructs of benefits to increasing calcium intake (*P* < 0.001), susceptibility to developing osteoporosis (*P* < 0.001), and self-efficacy regarding calcium (*P* < 0.001) [23]. This is inconsistent with the results of a study by Ahmadi- Tabatabei. In this study, the participants received educational material to improve their perceived capability regarding the benefits of doing physical activities and walking based on the TPB. According to the results, there was no significant difference in the score of activity between the two groups before the intervention. The difference remained insignificant after the intervention, and the score of physical activity did not reduce significantly after six weeks [32]. It is possible that the reason for the inconsistency between the results of the present with those reported Ahmadi-Tabatabi is that post-test was conducted two months after intervention in the present study, and it could be because the time and context for a complete change of behavior have not been consideredin the above study.

Independent sample t-test showed a significant difference in the knowledge score between the two groups after the intervention. The knowledge score increased significantly after the intervention in the intervention group (*p* < 0.05) (Table 2). This was in line with the results of studies by Pakpour Haji-Agha et al. and (28) Zhu et al. [33, 34]. Hatef Nia et al. also carried out a study on women working in pharmaceutical factories in Tehran. In this study, the educational program was presented directly (two 40–60 min educational sessions) and indirectly (pamphlet and handbook). The results showed a significant increase in the knowledge score three months after the intervention [35]. These studies are in line with the present study indicating the positive effect of the intervention and the need for educational interventions designed to improve preventive behaviors.

The results of independent sample t-test showed a significant difference in attitude between the two groups after the intervention, indicating that the educational program caused a significant increase in attitude in the intervention group (*p* < 0.05). This finding was consistent with the results of studies conducted by Shakeri Nejad et al. [36] and Kim et al. [37]. This finding was also in line with a study by Jangi et al. (2018) in which the effect of an educational intervention based on TPB on improving nutritional behaviors of high school students in the prevention of osteoporosis was investigated. The results showed the positive effect of this intervention on the partcipants’ attitude. There was no significant difference in the score of attitude between the three groups before the intervention. However, the score of attitude increased significantly in two intervention groups after the intervention (*p* < 0.05) while no significant change was observed in the control group [38].

The results of the present study showed a significant difference in the score of subjective norms between control and intervention groups after the intervention (*p* < 0.05) indicating that the educational intervention caused a significant improvement in subjective norms. Therefore, according to the results, implementing educational interventions and using influential people have positive effects on middle-aged individuals’ subjective norms regarding osteoporosis preventive behavior. This is in line with the findings of a study by Caron et al. in which an educational program was designed in nine months of school time and the results indicated a significant difference two months after the intervention [39]. Jangi et al. (2018) also found similar results in the prevention of osteoporosis [38].

The results showed that a significant difference in perceived behavioral control between control and intervention groups after the intervention (*p* < 0.05), indicating that perceived behavioral control about osteoporosis preventive behaviors increased significantly following the intervention. This finding was in line with the results of a study by Rusteaye Shalmayi et al. [40]. Duangpunmat et al. also conducted a study on individuals aged 35–59 years old who were prone to hypertension and found similar results [41]. The results showed no significant difference in osteoporosis preventive behavior between the intervention and control groups after the intervention (*P* > 0.05). This finding, however, was inconsistent with the results of studies by MohammediManesh et al. [42] and Juon et al. [43].

### Limitations

One of the limitations of the study was that the data were collected in a self-report manner using a questionnaire and it was likely that the answers were given in a positive or negative manner according to their mental status. Another limitation was the possibility of recall bias while completing the questionnaire. Furthermore one primary care center is selected to access the source population. However this center was the biggest center of the Kermanshah province and there was an acceptable diversity of the clients in terms of cultural and socio-economic status but selecting one primary care center can impact the results of this study in terms of external validity.

## Conclusions

The results of the present study showed that implementing an educational program that is compatible with culture, regional habits and context (despite controlling economic status) can help to improve knowledge, behavior, attitude, norms, behavioral control, and behavioral intention as well as preventive behaviors and decrease the factors and habits that cause osteoporosis. The findings of this study can be a basis for conducting further interventions considering the role of nurses in health-oriented education in all the community settings. For instance, nurses can use this study to obtain general important information regarding osteoporosis preventive behaviors and design and implement various educational programs to address the existing shortcomings.

## Data Availability

The datasets used and/or analyzed in the present study are available from the corresponding author on reasonable request.

## Abbreviations

TPB: Theory of Planned Behavior
WHO: World Health Organization
